# Interleukin-12 and -23 blockade mitigates elastase-induced abdominal aortic aneurysm

**DOI:** 10.1038/s41598-019-46909-y

**Published:** 2019-07-18

**Authors:** Huimin Yan, Ying Hu, Antonina Akk, Karen Ye, John Bacon, Christine T. N. Pham

**Affiliations:** 1 John Cochran VA Medical Center, Saint Louis, Missouri USA; 2 Department of Medicine, Division of Rheumatology, Washington University School of Medicine, Saint Louis, Missouri USA; 3 Department of Pathology and Immunology, Washington University School of Medicine, Saint Louis, Missouri USA

**Keywords:** Mouse, Aneurysm

## Abstract

Macrophages play an important role in the inflammatory process that contributes to the development of abdominal aortic aneurysm (AAA). Studies of human and mouse AAA tissue reveal expanded populations of macrophages producing an abundance of pro-inflammatory cytokines, including TNF-α, IL-12p40 and high level of metalloprotease 9 (MMP-9) at the late stages of disease. Herein, we show that blockade of IL-12p40 in the early phase of aneurysm development suppresses macrophage expansion, inflammatory cytokine and MMP-9 production and mitigates AAA development. Since IL-12 and IL-23 are related cytokines that share the common p40 subunit, we also evaluate the effect of direct IL-23 blockade on the development of AAA. Specific IL-23p19 blockade prevents AAA progression with the same efficiency as IL-12p40 antagonism, suggesting that the efficacy of anti-IL-12p40 treatment may reflect IL-23 blockade. IL-12p40 and IL-23p19 are also abundantly expressed in human AAA tissue. Our findings have potential translational value since IL-12p40 and IL-23p19 antagonists already exist as FDA-approved therapeutics for various chronic inflammatory conditions.

## Introduction

Abdominal aortic aneurysm (AAA) is a common and progressive vascular condition in elderly males that is associated with high risk of mortality due to rupture if left untreated. Although surgical repair is recommended for large aneurysms, medical therapy for small aneurysms is still lacking due to our incomplete understanding of the molecular mechanisms underlying the formation and progression of small AAA^1^, highlighting the need to further explore and validate candidate therapeutic targets.

Human AAA tissue and animal models suggest that AAA is an inflammatory disease characterized by transmural infiltration of the aortic wall with every type of leukocytes^2,3^. There is accumulating evidence that macrophages are an important source of matrix-degrading proteases that help to degrade the extracellular matrix (ECM)^4,5^. The role of metalloproteinases (MMPs) in preclinical models of AAA is well established^5,6^. And MMPs are expressed in increased amounts in human AAA^7,8^. More recent studies suggest that genetic polymorphisms of MMPs are associated with vascular dilation or aneurysmal disease^9–11^. Whether these proteases are directly responsible for the weakening and dilatation of the aortic wall remains a matter of debate^12^. Moreover, pharmacologic inhibition of MMP activity has not consistently delayed the progression of AAA in clinical trials^13^.

Macrophages also release a wide range of pro-inflammatory cytokines that may be involved in accelerating the growth of AAA^14^. IL-12 and IL-23 are related cytokines that share the same p40 subunit; however, the p19 subunit is specific to IL-23^15^. IL-12 and IL-23 direct the differentiation and expansion of Th1 and Th17 cells, respectively^16^. Animal studies show that mice deficient in the expression of the common p40 subunit of IL-12 and IL-23 are protected from developing many inflammatory and immune-mediated conditions^17–19^. IL-12/IL-23 have been indirectly implicated in AAA^20–22^. Sharma *et al*. found that genetic disruption of IL-23 attenuated elastase-induced AAA, likely through the suppression of IL-17 expression^23^. Thus, pharmacological modulation of IL-12/IL-23 may represent an attractive treatment strategy to interrupt the progression of AAA.

## Results

### Elastase-induced AAA model

**Figure 1:**
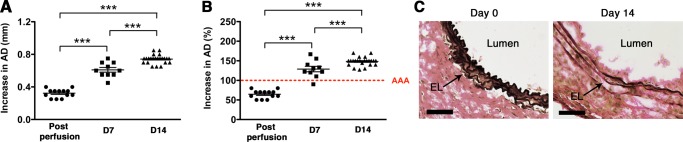
Transient elastase perfusion induces AAA. Mice were transiently perfused with elastase on day 0 and increase in aortic diameter (AD) was measured immediately post-perfusion, and on day 7 or day 14. Elastase perfusion resulted in ∼70% increase in AD on day 0 (post perfusion). AAA was defined as an increase in AD of greater than 100% on day 14 compared with the AD measured prior to elastase perfusion. AAA is expressed as the increase in AD in mm (**A**) or % (**B**). AAA was accompanied by elastic fiber degradation on day 14 (**C**). Scale bar = 250 um.

We used the well-established elastase-induced AAA model in which transient porcine elastase perfusion (for 5 min) of the infrarenal abdominal aorta on day 0 leads to aneurysmal dilatation at day 14^24^. AAA is defined as an increase in the aortic diameter (AD) of more than 100% over the pre-elastase perfusion measurements^24^. Elastase perfusion led to an immediate increase in AD of ~70% (Fig. 1A)^25^. WT C57BL/6 mice uniformly developed AAA (mean increase in AD of 148% or 0.74 mm) (Fig. 1A,B). AAA development also led to the characteristic elastic fiber fragmentation (Fig. 1C).

### Elastase-induced AAA leads to macrophage expansion

**Figure 2:**
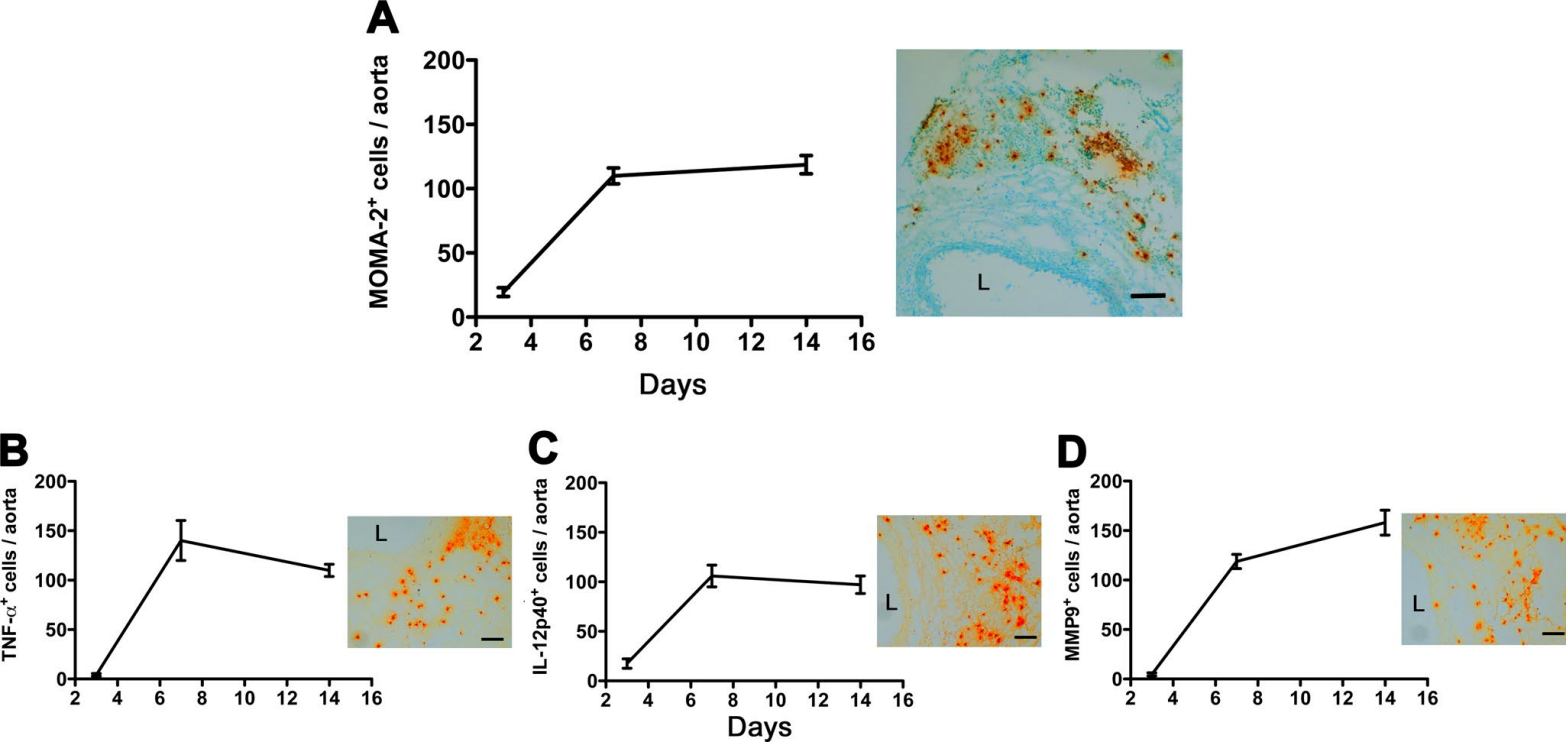
Macrophage expansion during AAA development. Mice were transiently perfused with elastase on day 0 and their aortas were harvested on days 3, 7, and 14. Aortic sections were stained for macrophages (MOMA-2) (**A**), TNF-α (**B**), IL12p40) (**C**), and MMP-9 (**D**). Scale bar 100 um. Values represent mean ± SEM, n = 6–8 aortas per time point. Values represent mean ± SEM, n = 6–8 aortas time point. ***p < 0.001.

To examine aortic wall macrophage accumulation during AAA progression, we performed a temporal analysis of macrophage number at different stages of disease post-elastase perfusion (days 3, 7, and 14). We found that macrophages (MOMA-2^+^ cells) began to accumulate as early as day 3-post elastase perfusion; the number of MOMA-2^+^ cells in the aortic wall continued to increase from day 3 to day 7 and remained elevated until day 14 (Fig. 2A). In parallel with the temporal expansion in MOMA-2^+^ cells, we found that the number of cells expressing inflammatory cytokines TNF-α and IL-12p40 peaked at day 7 and remained elevated at day 14 (Fig. 2B,C) while the number of cells expressing MMP-9 rose steadily, peaking on day 14 (Fig. 2D).

### IL-12 drives the inflammatory responses in elastase-induced AAA

**Figure 3:**
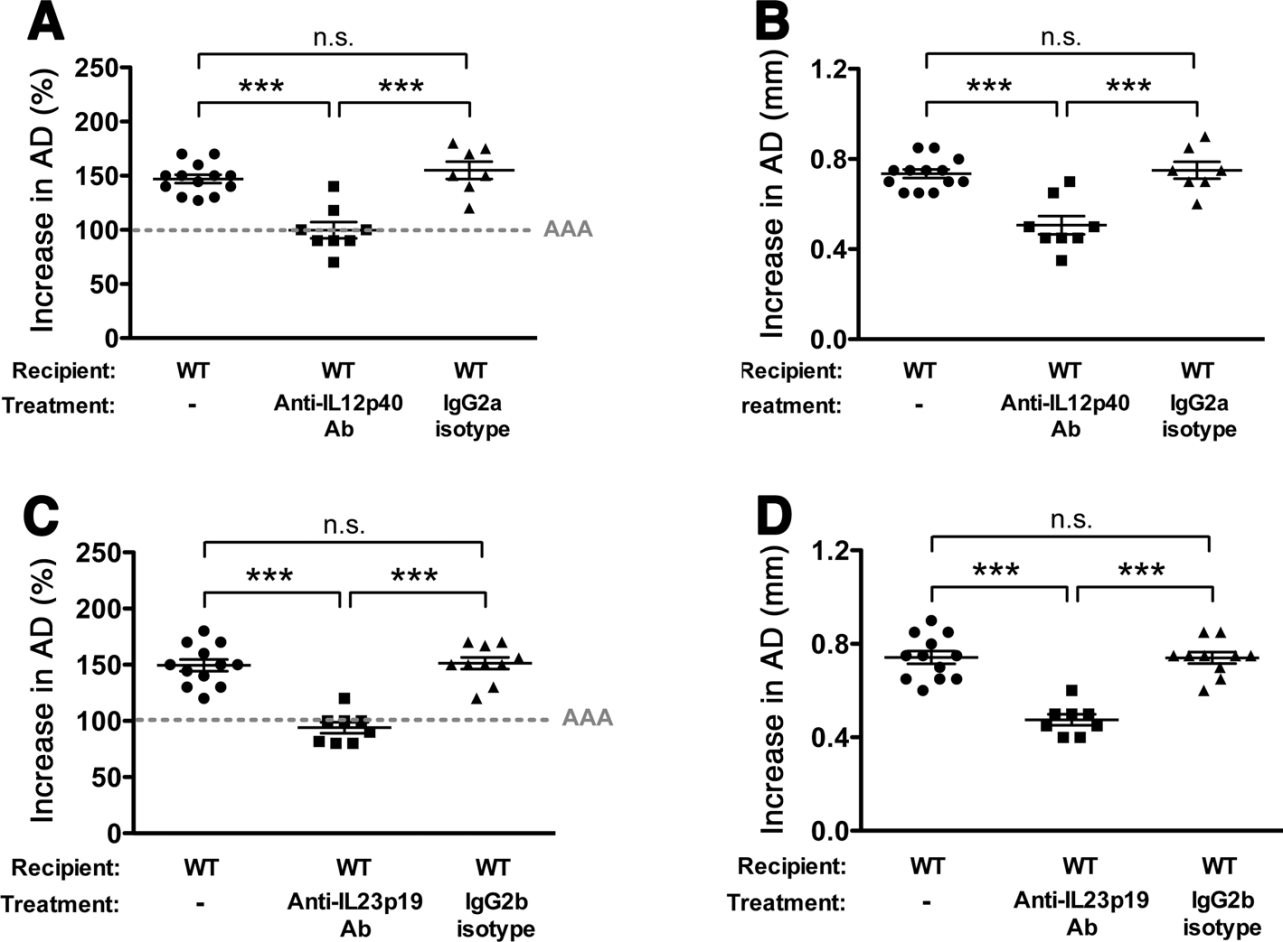
Antagonism of IL-12p40/IL-23p19 attenuates AAA development. Mice were perfused with elastase on day 0 and injected with anti-IL-12p40 or anti-IL-23p19 or isotype control (250ug) on days 3 and 8. AD was assessed on day 14. AAA is expressed as the % increase in AD (**A**,**C**) or the change in AD in mm (**B**,**D**). Values represent mean ± SEM, n = 7–8 mice per treatment. **p < 0.01, ***p < 0.001.

IL-12 is mainly known as a pro-inflammatory cytokine produced by activated antigen-presenting cells that induces Th1 differentiation, promotes IFN-γ production and acts as a link between innate and adaptive immunity^17^. Examination of AAA aortic wall tissue post-elastase perfusion, however, revealed that the majority of IL-12p40 secreting cells were from infiltrating MOMA-2^+^ cells (~70%) (Fig. S1), suggesting macrophages as the main source of IL-12p40. A recent study shows that IL-12 can “reprogram” macrophage phenotype and activity^26^, suggesting that modulation of the IL-12 axis may be beneficial to explore as a treatment strategy to interrupt the progression of AAA. To further understand the role of IL-12 in the progression of elastase-induced AAA, we administered a neutralizing monoclonal antibody (Ab) against the p40 subunit of IL-12 on day 3 and day 8-post elastase perfusion. We found that antagonism of IL-12p40 in the early stages of aneurysm development significantly attenuated AAA (increase in AD = 100% or 0.51 mm, n = 8, SD = 21% or 0.11 mm in the IL-12p40 antagonist treatment group versus AD = 155% or 0.750 mm, n = 7, SD = 21% or 0.10 mm in animals administered IgG isotype control, *p* < 0.001) (Fig. 3A,B). IL-12p40 antagonism significantly suppressed macrophage expansion (Fig. S2), and dampened the production of several macrophage-associated inflammatory mediators, including IL-12p40, IL-6, TNFα, and MMP-9 (Fig. S2) (*p* < 0.001 compared with isotype control). Moreover, we observed a marked reduction in the number of IL-17A^+^ cells in aortic wall tissue with IL-12p40 blockade *(p* < 0.001) (Fig. S2).

### IL-23 blockade mitigates elastase-induced AAA

IL-17A is an inflammatory cytokine produced by T helper cells in response to IL-23 signaling^16^. A previous study suggesting that IL-23 deficiency significantly attenuates elastase-induced AAA, perhaps through the modulation of IL-17 producing T helper cells (Th17)^23^, prompted us to evaluate the effect of direct IL-23 blockade on the development of AAA. Using a blocking Ab specific for the p19 subunit of IL-23, we demonstrated that anti-IL-23p19 treatment significantly dampened the development of AAA (increase in AD = 94% or 0.48 mm, n = 8, SD = 14% or 0.07 mm in the IL-23p19 antagonist treatment group versus AD = 151% or 0.74 mm, n = 10, SD = 16% or 0.08 mm in the isotype control group, *p* < 0.001) (Fig. 3B,C). Anti-IL-23p19 treatment prevented the onset and expansion of AAA with the same efficiency as IL-12p40 antagonism, suggesting that the efficacy of anti-IL-12p40 treatment may reflect IL-23 inhibition.

**Figure 4:**
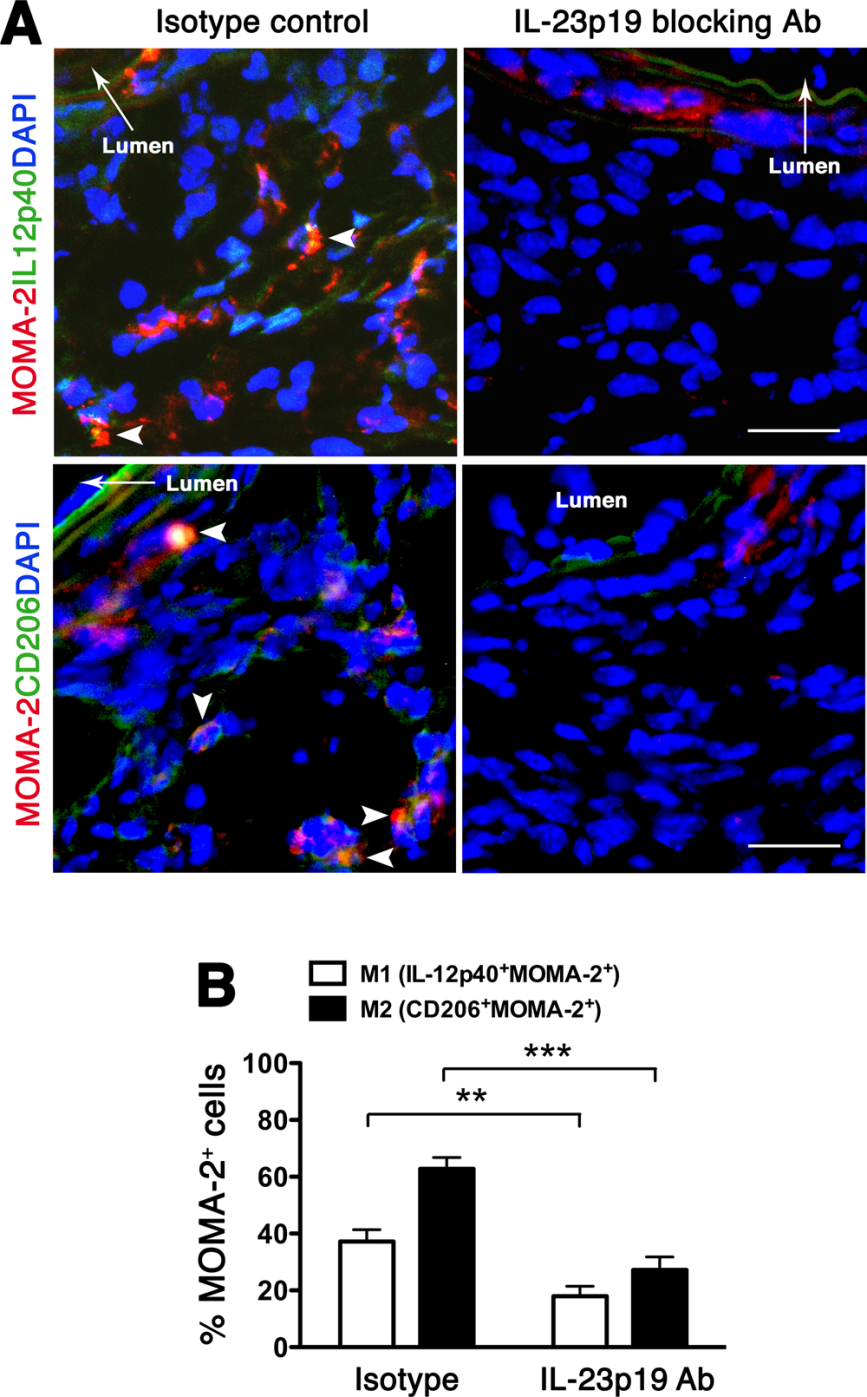
IL-23p19 blockade suppresses macrophage polarization. (**A**) Aortic sections obtained on day14 were double-stained for MOMA-2 (red) and IL12p40 (green) for M1 and CD206 (green) for M2. DAPI (blue) stained nuclei. Arrows indicate co-localization (yellow). Photographs are representative of 6–8 aortas per treatment. Scale bar = 25 um. (**B**) Enumeration of M1/M2 on day 14. Values represent mean ± SEM, n = 6–8 mice per treatment. **p < 0.01, ***p < 0.001.

**Figure 5:**
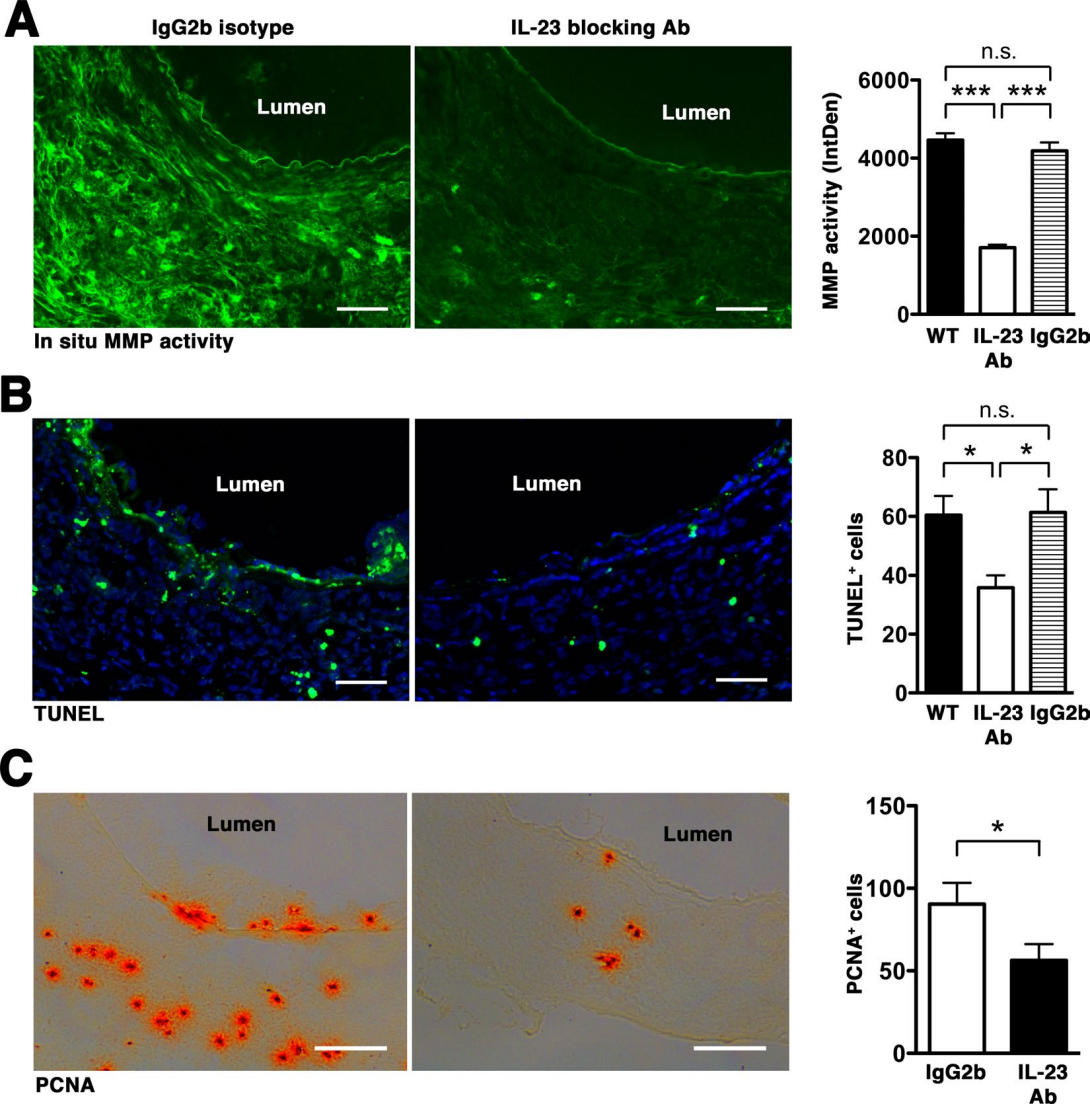
IL-23p19 blockade suppresses MMP activity and apoptotic cell death. Aortic sections obtained on day 14 were used to detect gelatinolytic activity of MMP by *in situ* zymography (**A**) apoptotic cells (TUNEL^+^, **B)**, cell proliferation (PCNA^+^, **C**). Scale bar = 50 um (**A**, **B**), 100 um (**C**). Values represent mean ± SEM, n = 4–6 sections per aorta, 6–8 aortas per treatment. *P < 0.05, **P < 0.001.

Macrophages have distinct phenotypes during AAA development with classically activated macrophages (M1) expressing pro-inflammatory cytokines while alternatively activated macrophages (M2) are involved in extracellular matrix remodeling and repair^27^. On day 14 post-elastase perfusion we found that aortic wall macrophages expressed both M1 (IL12p40) and M2 (CD206) markers but M2 macrophages were more predominant (Fig. 4). IL-23 blockade inhibited the expansion of macrophages and profoundly reduced the expression of macrophage-associated inflammatory mediators (Fig. S3). Likewise, IL-23 blockade mitigated T cell accumulation (Fig. S4) and IL-17A expression (Fig. S3). Double immunofluorescent staining showed that IL-23p19 blockade also inhibited macrophage polarization, resulting in significantly lower numbers of macrophages expressing either M1 or M2 markers (Fig. 4). IL-23p29 blockade also markedly suppressed the activity of MMPs, the main proteases involved in ECM remodeling (Fig. 5A), apoptotic cell death, as evidenced by decreased TUNEL staining (Fig. 5B), and cell proliferation, as detected by proliferating cell nuclear antigen (PCNA, Fig. 5C). These results suggest that IL-23p19 inhibition dampens the inflammatory responses and MMP expression, preserving the overall architecture of the aortic wall tissue and mitigating aneurysmal dilatation.

### IL-12/IL-23 axis is active in human AAA

**Figure 6:**
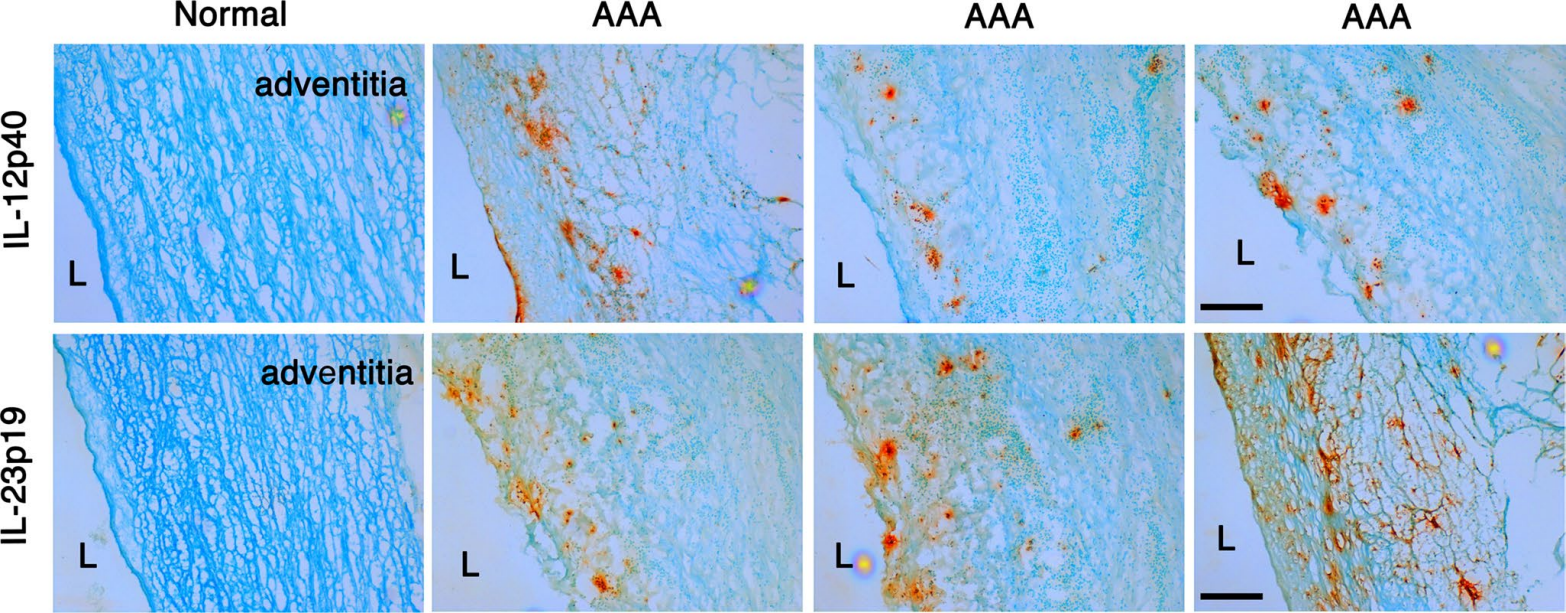
IL-12/IL-23 in human AAA tissues. Immunohistochemistry of human AAA tissues revealed abundant expression of IL-12p40 and IL-23p19 (orange) throughout all layers of aortic wall tissue. Normal aortic wall tissue showed no specific staining. N = 3 independent AAA samples. L = lumen. Scale bar = 200 um.

Next, we sought to determine whether IL-12 and IL-23 could represent relevant therapeutic targets in human AAA. Immunostaining of human AAA tissue (n = 3) for IL12p40 and IL-23p19 confirmed their abundant presence throughout all layers of the aortic wall while non-AAA tissue showed no specific staining (Fig. 6). The IL-23p19 immunostaining corroborates previous studies that examined *IL-23* gene expression level in aortic tissue from AAA patients^23^.

## Discussion

Careful analysis of aortic wall tissue at different time points post elastase perfusion shows that early stage elastase-induced AAA is associated with the infiltration of cells expressing pro-inflammatory cytokines such as TNF-α, IL-6 and IL-12p40. End-stage experimental aneurysm is associated with a shift towards cells that express high level of MMP-9, which may trigger substantial collateral tissue damage when released, further driving the aneurysmal phenotype. In an oversimplification, macrophages can be divided into two populations, the classically and alternatively activated macrophages. Classically activated macrophages (M1) are generated through IFN-γ and TNF-α stimulation and secrete high levels of pro-inflammatory cytokines (TNF-α, IL-1β, IL-6, IL-12) and nitric oxide synthase (NOS). Alternatively activated macrophages (M2) are induced *in vitro* by IL-4; they promote matrix reorganization and wound healing^27^. Animal models of AAA suggest that M1 macrophages predominate in early stages of aneurysm development while M2 macrophages are more abundant at late-stage disease^28,29^. Macrophages are a highly heterogeneous cell population that can rapidly switch their functional phenotype in response to local microenvironmental signals^30^. And while the definition of M1 and M2 macrophages is well delineated *in vitro*, their functional distinction and phenotypes *in vivo* are less clear. Antagonism of IL-23p19 is associated with decreases in both M1 and M2 macrophages, which suggests that the higher M2/M1 macrophage ratio observed in late-stage elastase-induced AAA likely represents an effort to control and repair the aortic wall ECM in response to the pro-inflammatory activities exerted by expansion of M1 macrophages. IL-12 and IL-23 are also important drivers of T cell differentiation and proliferation. While IL-12 (and IFN-γ) promotes Th1 differentiation, IL-23 drives the development of Th17 cells^31^. Antagonism of IL-12p40 and IL-23p19 not only suppressed the expansion of macrophages but also inhibited T cell proliferation, thus abrogating the Th1/Th17-driven inflammatory responses during elastase-induced AAA development.

The discovery that IL-12 and IL-23 are active participants in the pathogenesis of several immune-mediated diseases have prompted the development of therapeutic agents targeting these cytokines or their receptors. Ustekinumab, the first monoclonal antibody directed against the p40 subunit of IL-12 and IL-23 is approved for the treatment of psoriasis, psoriatic arthritis, and Crohn’s disease^16,32,33^. Likewise, therapeutic agents targeting IL-23p19 have recently been approved or are in late phase clinical trials for the treatment of psoriasis and Crohn’s disease^34–36^. Although a previous study finds that genetic disruption of IL-23 attenuated elastase-induced AAA^23^, our key finding herein is that therapeutic blockade of the IL-12/IL-23 axis effectively suppresses AAA progression when given in the early phase of aneurysm development. On the other hand our results also seemingly contradict findings by Sharma *et al*. suggesting that deficiency of IL-12p40 promotes angiotensin II (Ang II)-induced AAA^37^. The discrepancy may be explained by a complete loss of IL-12p40 from birth, which may directly impact the polarization of macrophages in response to various levels of inflammatory modulators at different stages of disease progression.

The role of IL-17 in AAA also remains controversial. A previous study suggests that IL-17 is a critical mediator of elastase-induced AAA and genetic deletion of IL-17 not only protects mice against AAA formation but also leads to significant reduction of several inflammatory cytokines (TNF-α, IFN-γ and MCP-1), indicating that IL-17 is an upstream mediator of the inflammatory cascade^23^. These results corroborate findings in another study that uses digoxin to attenuate Ang II-induced and elastase-induced AAA, showing that IL-17A related inflammatory responses were suppressed in a dose-dependent manner^38^. In addition, IL-17 is implicated in the Ang II-induced aortic dissections^39^. On the other hand, Romain *et al*. demonstrate that overexpression of suppressor of cytokine signaling 3 (SOCS3) significantly decreases IL-17 expression and markedly increases aneurysm severity in a model of Ang II-induced AAA, leading the investigators to suggest that IL-17 has a protective role in this process^40^. It is possible that overexpression of SOCS3 modulates the expression of other proinflammatory mediators in Ang II-induced AAA and the indirect decrease in IL-17 is merely coincidental and not causal. Our data herein agree with previous studies suggesting a pro-inflammatory role for IL-17.

In summary, there is currently no drug therapy for AAA. We posit that blockade of IL-12/IL-23 axis as treatment strategy to halt the progression of small aneurysms merits further consideration given the availability of the immunotherapeutics in the clinic combined with the observation that IL-12p40 and IL-23p19 are abundantly expressed in human AAA tissue.

## Methods

### Animals

WT C57BL/6J mice (Cat# 000664) were obtained from the Jackson Laboratory (Bar Harbor, ME). All mice were kept in a standard pathogen-free environment at Washington University Specialized Research Facility (St. Louis, MO). All animal experiments were performed in accordance to guidelines and protocols approved by the Division of Comparative Medicine at Washington University in St. Louis. The animal protocol is subjected to annual review and approval by The Animal Studies Committee of Washington University.

### Human tissues

All research involving human tissues was performed in accordance with relevant guidelines and regulations established by the Washington University in St. Louis Institutional Review Board (IRB) Committee. De-identified human abdominal aorta tissue specimens were provided by Dr. Robert Thompson who obtained them from discarded aortic aneurysm tissues harvested at time of elective surgery through a protocol approved by the Institutional Review Board at Washington University School of Medicine. All participants provided written informed consent. The aortic tissues were snap-frozen, embedded in OCT and stored at −80 °C until use.

### Murine elastase-induced AAA

AAA was induced as previously described^41^. Only male mice were used in these studies, as AAA is a disease with strong male predominance^42^. Briefly, 8- to 10-week-old male wild-type (WT) C57BL/6J mice were anesthetized with an i.p. injection of ketamine (87 mg kg^−1^), xylazine (13 mg kg^−1^), and acepromazine (2 mg kg^−1^) KXA cocktail. For post-op pain control mice were administered Buprenorphine-SR (0.5 mg kg^−1^) one hour prior to surgery and the effect was expected to last 72 h. Immediately prior to surgery, lidocaine (0.5%) was injected into the subcutaneous space below the planned incision line. A laparotomy was then performed under sterile conditions. The infrarenal aorta, from the left renal vein to the aortic bifurcation was isolated, ligated and perfused for 5 min and 30 sec with a solution containing 0.145 U/ml type 1 porcine pancreatic elastase (Cat# E-1250, Sigma-Aldrich, St Louis, MO) via infusion pump. The tie was released and the maximal post-perfusion aortic diameter was measured with a calibrated ocular grid and the aortotomy closed. On day 14, unless otherwise indicated, a second laparotomy was performed following anesthesia with KXA cocktail and the aortic diameter measured prior to euthanasia and tissue procurement.

In some studies WT mice were injected i.p. with rat anti mouse-IL-12p40 monoclonal antibody (250 ug on days 3 and 8, Cat# MAB4991, R&D, Minneapolis, MN) or mouse anti mouse-IL-23p19 monoclonal antibody (250 ug on days 3 and 8, Cat# 513806, Biolegend, San Diego, CA). Control groups received either purified rat IgG2a functional grade GOLD (250 ug on days 3 and 8, Cat# I-1177, Leinco Techologies, Fenton, MO) or purified mouse IgG 2b functional grade GOLD (250 ug on days 3 and 8, Cat# I-119, Leinco Techologies, Fenton, MO). The number of mice per treatment is indicated in the text and figure legends. Elastin staining was performed as previously described^43^.

### Immunofluorescence

The mouse aortas were harvested on days 7 or 14, embedded in OCT and sectioned. Cross sections of aortic tissues (9 µm) were fixed in 4% paraformaldehyde, blocked in 8% BSA in PBS and incubated with the following primary antibodies: rat anti-mouse MOMA-2 (1:200 dilution; Cat# ab33451, Abcam, Cambridge, United Kingdom), biotinylated-IL-12p40 (1:100 dilution; Cat# 505302, Biolegend, San Diego, CA), rat anti-mouse CD206 (1:200 dilution; Cat: MCA2235, Bio-Rad [Formerly AbD Serotec], Hercules, CA), or rabbit anti-mouse CD8a (1:200 dilution; Cat# bs-0648R, Bioss antibodies, Woburn, Massachusetts) followed by the appropriate rhodamine red- or FITC-conjugated secondary antibody (1:100–1:200; Jackson ImmunoResearch Laboratories, West Grove, PA) or Streptavidin Alexa Fluor 488 conjugate (1:400 dilution; Cat# S-11223, Molecular Probes, Eugene, OR). Nuclei were counterstained with DAPI. Images were acquired on a Leica DM 2000 microscope fitted with a Leica DMC 4500 color camera and analyzed with Leica Application Suite (LAS) X software. Single- or double-color images were loaded into ImageJ and cell enumeration was performed in a blinded fashion. Data were obtained from 3–4 non-overlapping fields per aortic cross-section, 6–9 sections per aorta, n = 6–8 aortas per time point.

### Immunohistochemistry

Immunohistochemical staining was performed on formalin-fixed, OCT-embedded 9 μm sections of mouse or human AAA tissue. Rat anti-mouse MOMA-2 (1:200 dilution; Cat# ab33451, Abcam, Cambridge, United Kingdom), rabbit anti-mouse TNF-α (1:100 dilution; Cat# ab34674, Abcam, Cambridge, United Kingdom), biotinylated anti-mouse IL-12p40(1:100 dilution; Cat# 505302, Biolegend, San Diego, CA), rabbit anti-mouse IL-6 (1:100 dilution; Cat# NB600-1131, Novus biologicals, Littleton, CO), rat anti-mouse MMP-9 (1:200 dilution; Cat# 116103, R&D, Minneapolis, MN), rat anti-mouse IL-17A (1:100 dilution; Cat# 560666, BD Pharmingen, San Jose, CA), biotinylated anti-PCNA (1:100 dilution; Cat# 307904, Biolegend), biotinylated anti-mouse CD3 (1:100 dilution; Cat# 553060, BD Pharmingen) primary antibodies were applied to the frozen cross-sections for 1 hour at RT followed by the appropriate HRP-conjugated secondary antibodies. Data presented was derived from six to nine serial cross-sections that spanned the entire abdominal aorta, with 5–8 aortas per time point. For human tissue, mouse anti-human IL-12p40 (1:100 dilution; Cat# 501801, Biolegend, San Diego, CA), rabbit anti-human IL-23p19 (1:100 dilution; Cat# 3793, ProSci, Poway, CA) antibodies were employed. Staining was performed on at least three independent sections per human AAA tissue samples (n = 3) and the pattern was confirmed on 3–6 independent tissue samples.

### *In situ* zymography

Non-fixed, frozen sections (9 μm) of day 14 aortas were incubated with a fluorogenic gelatin substrate (DQ gelatin at 25 μg/ml, Molecular Probes) for 1 h at room temperature. For negative control, slides were incubated in the presence of 25 mM EDTA. The specific removal of essential divalent cations resulted in no detectable gelatinolytic activity. Images were acquired on a Leica DM 2000 microscope fitted with a Leica DMC 4500 color camera and analyzed with Leica Application Suite (LAS) X software. Images were loaded into ImageJ for analysis. Using the brightness to filter the picture, positively stained areas or the regions of interest (ROIs) were isolated. The ROIs were measured and presented as integrated optical density (IntDen). Data represent 4–6 sections per aorta, 6–8 aortas per treatment.

### TUNEL assay

Detection of apoptotic cells was performed using *In situ* Cell Death Detection Kit, Fluorescein (Cat: 11-684-795-910, Roche) with the terminal deoxynucleotidyl transferase-mediated dUTP nick-end labeling (TUNEL) assay. Freshly prepared TUNEL reaction mixture were applied to aortic sections for 60 min at 37 °C, rinsed 3–5 times with PBS and mounted with VECTASHIELD mounting medium with DAPI (Cat: H-1200, Vector Laboratories). The TUNEL^+^ cells were enumerated across the entire aortic section. Data represent 4–6 sections per aorta, 6–8 aortas per treatment.

### Statistical analysis

Comparisons between two groups were performed by two-tailed, unpaired *t* test without correction. Comparisons between multiple groups (≥3) were performed by one-way ANOVA followed by Bonferroni’s posttest to compare all groups of data. F test was used to compare variances within each group of data and the difference in variances was found to be not significant between groups. Data are presented as the mean ± SEM. A *p* value < 0.05 was considered significant.

## Supplementary information

Supplementary information

## Data Availability

Data generated in this current study are available from the corresponding author on reasonable request.
