# Supplementary material for: Interleukin-12 and -23 blockade mitigates elastase-induced abdominal aortic aneurysm
Source: Sci Rep. 2019 Jul 18;9:10447. doi: 10.1038/s41598-019-46909-y (PMC6639297; doi:10.1038/s41598-019-46909-y)
Supplement: Supplementary file 1 — Supplementary information [file 41598_2019_46909_MOESM1_ESM.pdf]

Interleukin-12 and -23 blockade mitigates elastase-induced abdominal aortic aneurysm

Huimin Yan[1,2], Ying Hu[1,2], Antonina Akk[1], Karen Ye[1], John Bacon[1], and Christine T.N. Pham[1,2,3]

[1]John Cochran VA Medical Center, Saint Louis, Missouri USA

[2]Department of Medicine, Division of Rheumatology, Washington University School of Medicine, Saint Louis, Missouri, USA

[3]Department of Pathology and Immunology, Washington University School of Medicine, Saint Louis, Missouri, USA

Address correspondence to: Christine T.N. Pham, 660 South Euclid Avenue, Campus Box 8045, Saint Louis, MO 63110. Telephone: 314-362-9043. Fax: 314-454-1091. E-mail: cpham@wustl.edu

A

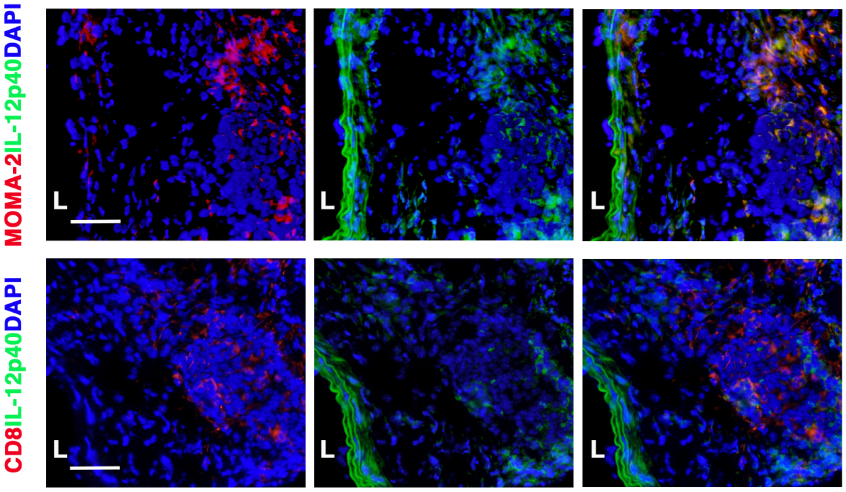

B

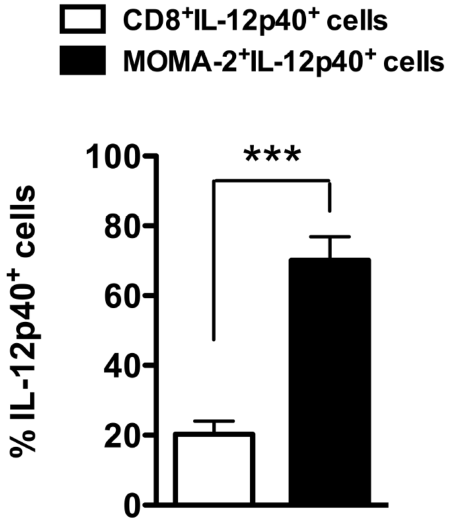

Figure S1. Macrophages represent the main source of IL-12p40. (**A**) Aortic tissue obtained from WT mice on day 14-post elastase perfusion were stained for IL-12p40 (green) in MOMA-2+ (red, upper row) or CD8+ cells (red, lower row). Scale bar = 50 μm. L = Lumen. DAPI (blue) stained nuclei. (**B**) Percent of MOMA-2+IL-12p40+ vs CD8+IL-12p40+ cells were calculated from 3-4 nonoverlapping fields per aortic cross-section, 6-9 sections per aorta, n=6-8 aortas. ***$p < 0.001$

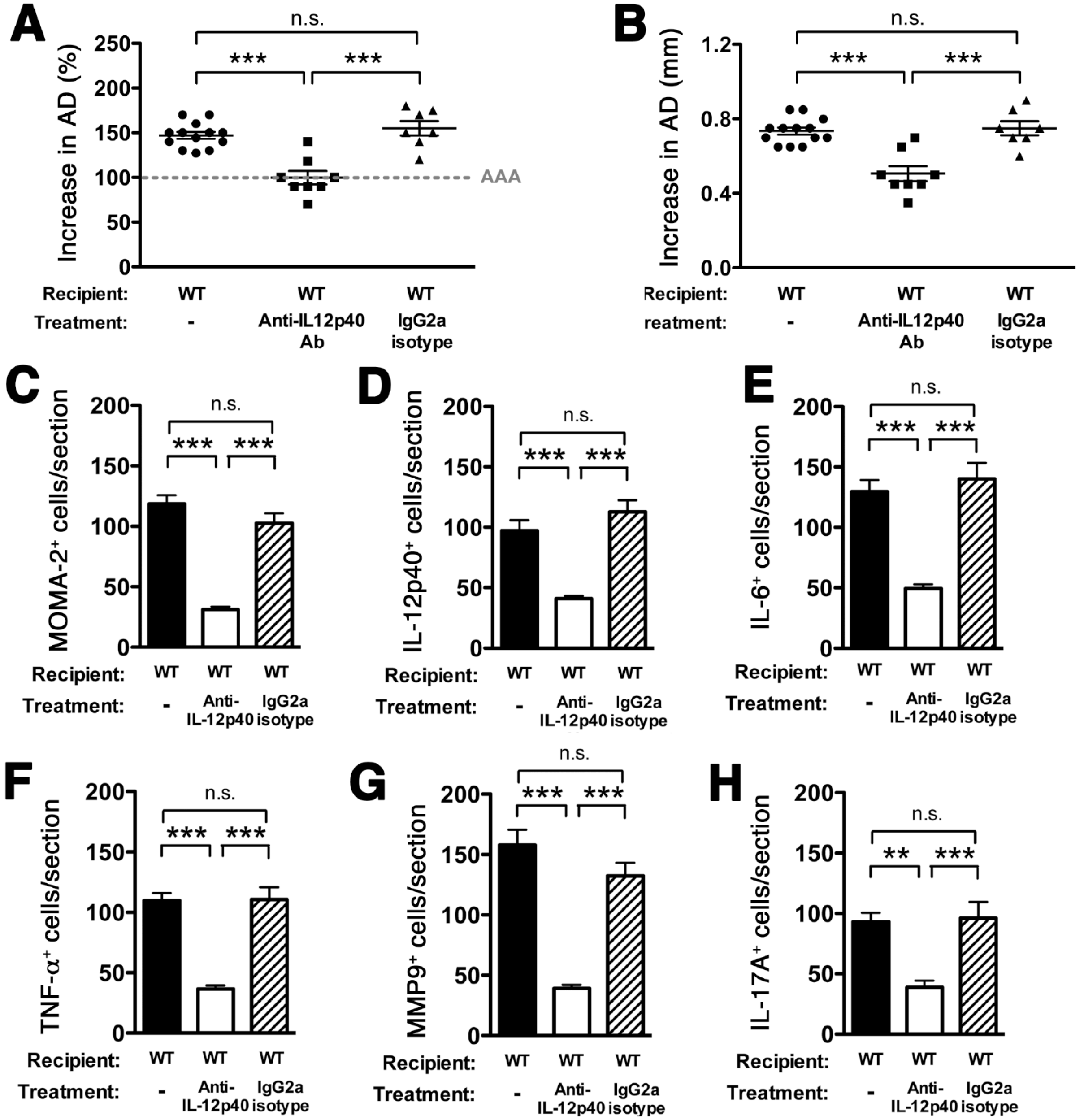

Figure S2. Antagonism of IL-12p40 attenuates AAA development. Mice were perfused with elastase on day 0 and injected with anti-IL-12p40 or isotype control (250ug) on days 3 and 8. AD was assessed on day 14. AAA is expressed as the % increase in AD (**A**) or the change in AD in mm (**B**). Antagonism of IL-12p40 attenuated macrophage expansion (**C**) and the expression of IL12p40, IL-6, and TNF-α, and MMP9 (**D-G**). IL-12p40 antagonism also attenuated the expression of IL-17A (**H**). Values represent mean $\pm$ SEM, n = 7-8 mice per treatment. $^{**}p < 0.01$, $^{***}p < 0.001$

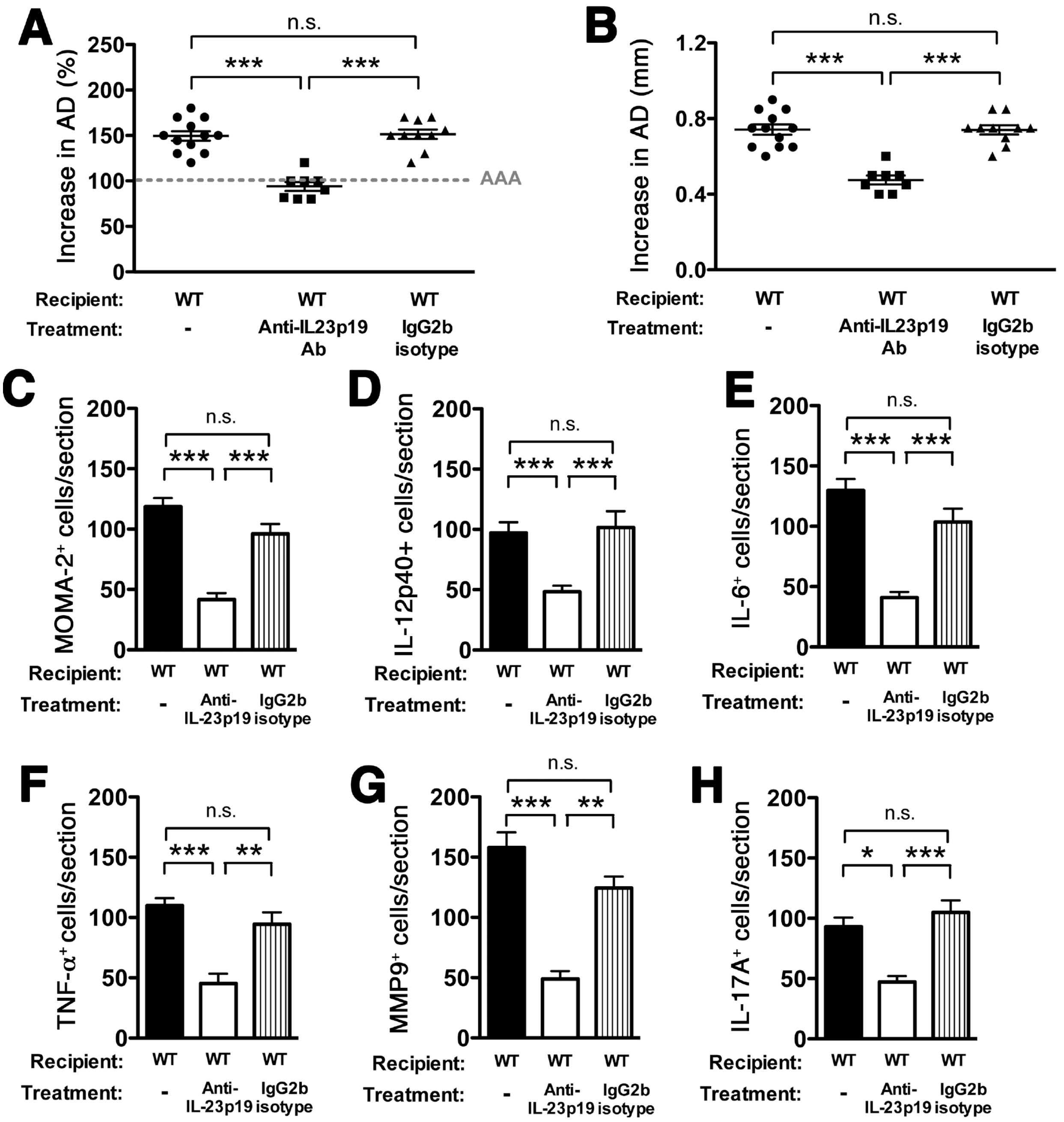

Figure S3. Antagonism of IL-23p19 attenuates AAA development. Mice were perfused with elastase on day 0 and injected with anti-IL-23p19 or isotype control (250ug) on days 3 and 8. AD was assessed on day 14. AAA is expressed as the % increase in AD (**A**) or the change in AD in mm (**B**). Antagonism of IL-23p19 attenuated macrophage expansion (**C**) and the expression of IL12p40, IL-6, TNF-α, and MMP9 and IL-17A expression (**D-H**). Values represent mean $\pm$ SEM, n = 8-10 mice per treatment. $^{**}p < 0.01$, $^{***}p < 0.001$

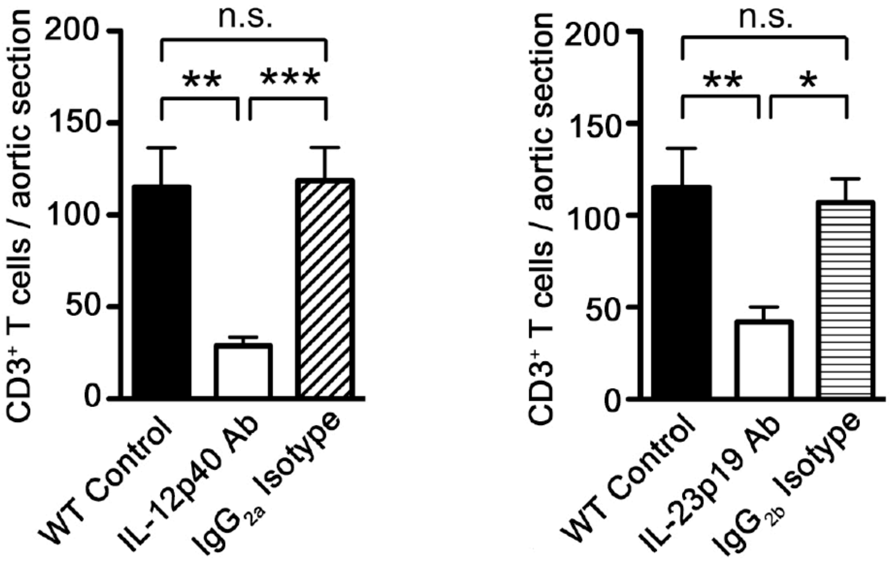

Figure S4. Antagonism of IL-12p40 and IL-23p19 suppresses T cell proliferation. Mice were perfused with elastase on day 0 and injected with anti-IL-12p40 or IL-23p19 or isotype control (250ug) on days 3 and 8. $CD3^+$ T cells were enumerated on day 14. Values represent mean ± SEM, 6-9 sections per aorta, n=6-8 aortas. *p < 0.05, **p < 0.01, ***p < 0.001
